# Comparative Plasticity Responses of Stable Isotopes of Carbon (δ^13^C) and Nitrogen (δ^15^N), Ion Homeostasis and Yield Attributes in Barley Exposed to Saline Environment

**DOI:** 10.3390/plants11111516

**Published:** 2022-06-05

**Authors:** Muhammad Iftikhar Hussain, Zafar Iqbal Khan, Taimoor Hassan Farooq, Dunia A. Al Farraj, Mohamed Soliman Elshikh

**Affiliations:** 1 Department of Plant Biology & Soil Science, Universidade de Vigo, Campus As Lagoas Marcosende, 36310 Vigo, Spain; 2 Research Institute of Science and Engineering, University of Sharjah, Sharjah P.O. Box 27272, United Arab Emirates; 3 Department of Botany, University of Sargodha, Sargodha 40100, Pakistan; zafar.khan@uos.edu.pk; 4 Bangor College China, A Joint Unit of Bangor University and Central South University of Forestry and Technology, Changsha 410004, China; taimoorhassan2055@gmail.com; 5 Department of Botany and Microbiology, College of Science, King Saud University, P.O. Box 2455, Riyadh 11451, Saudi Arabia; dfarraj@ksu.edu.sa (D.A.A.F.); melshikh@ksu.edu.sa (M.S.E.)

**Keywords:** *Hordeum vulgare*, stable isotope composition of carbon and nitrogen, saline water stress, isotope ecology, yield stability, ion homeostasis

## Abstract

Salinity is a major threat to agricultural productivity worldwide. The selection and evaluation of crop varieties that can tolerate salt stress are the main components for the rehabilitation of salt-degraded marginal soils. A field experiment was conducted to evaluate salinity tolerance potential, growth performance, carbon (δ^13^C) and nitrogen isotope composition (δ^15^N), intrinsic water use efficiency (iWUE), harvest index, and yield stability attributes in six barley genotypes (113/1B, 59/3A, N1-10, N1-29, Barjouj, Alanda01) at three salinity levels (0, 7, and 14 dS m^−1^). The number of spikes m^−2^ was highest in Alanda01 (620.8) while the lowest (556.2) was exhibited by Barjouj. Alanda01 produced the highest grain yield (3.96 t ha^−1^), while the lowest yield was obtained in 59/3A (2.31 t ha^−1^). Genotypes 113/1B, Barjouj, and Alanda01 demonstrate the highest negative δ^13^C values (−27.10‰, −26.49‰, −26.45‰), while the lowest values were obtained in N1-29 (−21.63‰) under salt stress. The δ^15^N was increased (4.93‰ and 4.59‰) after 7 and 14 dS m^−1^ as compared to control (3.12‰). The iWUE was higher in N1-29 (144.5) and N1-10 (131.8), while lowest in Barjouj (81.4). Grain protein contents were higher in 113/1B and Barjouj than other genotypes. We concluded that salt tolerant barley genotypes can be cultivated in saline marginal soils for food and nutrition security and can help in the rehabilitation of marginal lands.

## 1. Introduction

Global agriculture is unable to cope with the existing climate change scenario and to feed the worlds growing population that is projected to increase from 6.7 billion (2005) to 9.2 billion by 2050 [1]. Among all these anthropogenic factors, drought, salinity, and climate change are the principal players behind the land degradation and desertification leading to a significant reduction in crop production and yield decline [2,3,4,5]. Due to the scarce water resources and drought episodes, the irrigation water requirement in Arabian Gulf countries is mostly fulfilled through salty ground water and treated wastewater that is recruited to irrigate a significant land area (forestry, landscaping, roadside plantation) [5]. To meet the growing need of agriculture, date palm fruit gardens and landscaping, the Gulf countries are using desalinated water (7.2%) and groundwater (91%) to meet their requirements [6]. In this context, appropriate crop accessions that can be well adapted to the marginalized lands and available non-conventional water resources are suitable options for long-term rehabilitation and desertification resistance [3,7,8,9,10].

Barley is an important grain crop and ranked fourth among the cereal crops after wheat, rice, and maize [11]. It is mainly used as food, animal fodder, and as a raw material for beer production [12]. Several authors have demonstrated that barley can tolerate a number of environmental stresses, such as drought [13,14], salinity [15], and heavy metals [16]. However, salt tolerance within genotypes of barley under field conditions has not been evaluated intensively. Therefore, the study of genetic diversity and phenotypic plasticity should be integrated in order to evaluate and select the most tolerant genotypes within a wide range of salinity among this plant species. Furthermore, the growth, yield, and productivity of barley are highly variable in the Middle East and North Africa region because the local cultivars do not have sufficient tolerance potential against prevailing environmental constraints, especially drought and salinity. Most researchers have evaluated the variation in salinity tolerance using growth chamber or green house at a single level of salinity and there was no validation of those results under the field setting. Meanwhile, studies conducted in a controlled growth chamber generally involve the determination of salinity stress on seeding growth over a short period of time (often 1–7 days), which does not correspond to salt stress in the field that might indicate a wide variation in the growth, development, physiological, and yield traits [17].

It has been observed that among the population of particular crop genotypes, wide variation exists at various growth and development stages for salinity tolerance. However, it was difficult to predict which salinity range will be appropriate for the screening, selection, and evaluation of genotypes that can best correlate with genetic diversity under field conditions. This kind of study is very important in order to develop efficient breeding programs and tool kits of salt tolerant crop genotypes and to assess the growth, physiological, and yield traits under field conditions [18]. Efforts to enhance crop yields under salinity stress have also had limited success because the underlying mechanisms of salt tolerance have not been turned into useful selection criteria to evaluate a wide range of phenotypic plasticity and genotypes. Several authors have studied the salinity tolerance potential among a wide range of crop plants at the germination and seedling growth stages and showed a large genetic difference among them [19,20]. However, little attention has been paid to show a correlation regarding this early evaluation of salinity tolerance at germination with field condition [21]. However, it is worthwhile to mention that these authors made significant efforts to explain the Na^+^ exclusion, K^+^ accumulation, and K^+^/Na^+^ as reliable indicators for selecting suitable genotypes that can tolerate soil and irrigation water salinity [22]. The success of dual-purpose barley in marginal environments is subject to proper agronomic management practices along with the use of improved genotypes.

It is an urgent task of agronomists, plant physiologists, and plant breeders to identify and evaluate the genotypes and plant phenotypic plasticity using non-invasive, rapid, and reliable methods in order to screen the desired traits in a particular environment. The evaluation of the salinity tolerance potential of different genotypes and plant phenotypic attributes is highly necessary in order to understand physiological responses of the target genotypes and concerned traits associated with them [23,24]. The present situation can be changed through the introduction of new salt tolerant and higher yielding barley genotypes that have good yield stability and better salt tolerance potential. This will help to conserve freshwater resources as well as economic and ecological benefits for the sustainable development of salt-degraded marginal lands [6,25,26]. It is important to screen, select, and evaluate the large collection of barley genotypes to check their performance (growth, yield stability, physiological characteristics) and traits are suited to salinity tolerance under field condition. In the present field study, a set of 28 genotypes from a previous trial [27,28,29] were selected for elucidating the performance of different agronomical attributes (growth, number of tillers, plant biomass), yield traits (number of spikes, number of grains/spike, grain yield, harvest index), and biochemical attributes (Na^+^, Cl^−^, K^+^), to find more suitable and tolerant genotypes under sandy marginal lands. The current study will provide a basis to promote barley cultivation on a large scale in the salt affected agro-ecosystem environment of the UAE. In addition, genotypes that showed stable yield and salt-tolerance potential will be included in the barley breeding programs for the development and release of salt-tolerant cultivars for seed multiplication and distribution among NARS for multi-location testing and large-scale cultivation.

The phenotypic plasticity, genotype variability, and agronomic adaptation of barley are extremely wide and vary significantly from hot arid to subtropical humid climates. Barley batini land races have not been characterized for salt tolerance on morphological, biochemical, ecophysiological, and isotopic bases. The main aim of the present study was the evaluation of batini barley land races and genotypes through the elucidation of salinity tolerance potential, growth performance, leaf ion homeostasis, leaf carbon and nitrogen isotope discrimination, intrinsic water use efficiency, harvest index, and yield stability attributes on six barley genotypes (113/1B, 59/3A, N1-10, N1-29, Barjouj, Alanda01) at three salinity levels (0, 7 and 14 dS m^−1^). For this study, it was hypothesized that batini barley land races and genotypes are genetically diverse and vary for salt tolerance potential. The evaluation of the plasticity of physiological attributes, such as number of tillers/m^2^, fresh biomass (FW), dry biomass (DW), grain yield, harvest index, and leaf Na^+^, K^+^, and Cl^−^ concentration, leaf carbon and nitrogen isotope discrimination, and intrinsic water use efficiency, may help to develop a better understanding of mechanisms of salt tolerance.

## 2. Materials and Methods

### 2.1. Experiment Site and Climatic Conditions

**Figure 1:**
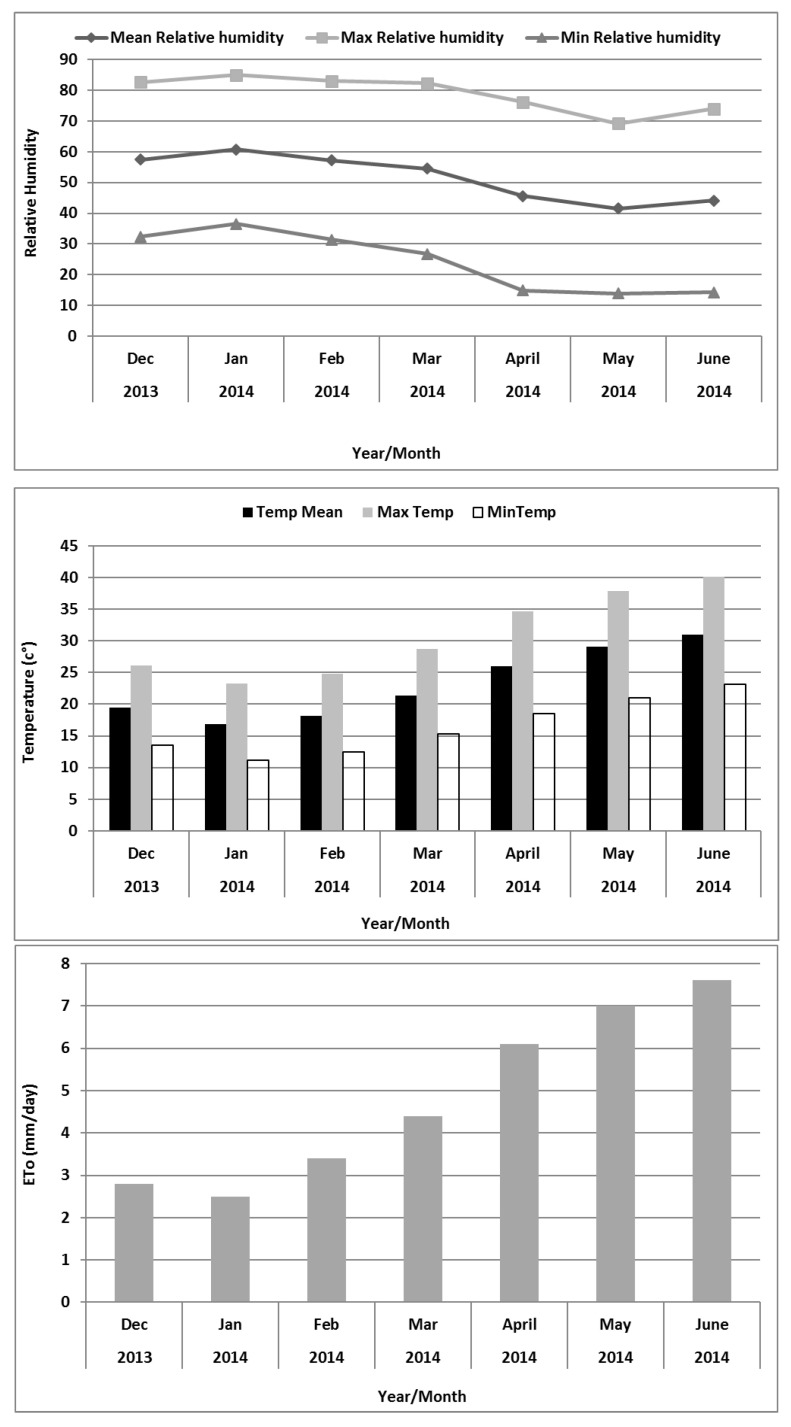
Monthly average values of mean (T mean), maximum (T maxi), and minimum (T min) air temperature and reference evapotranspiration (ETo) in the ICBA weather station, Dubai, UAE from December 2013 to June 2014.

The field trials were conducted at an agriculture experiment research station (ICBA, Dubai, UAE) from December 2013 to May 2014. The site is located at N 25°05.847; E 055° 23.464. The experimental field was nutrient-poor, sandy soil (sand 98%, silt 1%, and clay 1%), calcareous (50–60% CaCO_3_), porous (45% porosity), and moderately alkaline (pH 8.22). The electrical conductivity of saturated extract (Ec) is 1.2 dS m^−1^ and the soil has good drainage capacity and is classified as carbonatic, hyperthermic typic, and torripsamment. To keep the area drained and to control soil salinization at the experimental station, a sub-surface drainage system is installed at 2 m depth from the soil surface. From December to February, the temperature is significantly lower, days are cooler and dry (10 °C, temperature at night), while during the summer season (April to October), the temperature is high, can reach up to 50 °C, and the climate is extremely hot and dry with lots of humidity. During summer, there is almost no chance of rainfall and the sky is mostly cloudless. Average annual temperature, rainfall, and humidity are shown in Figure 1.

### 2.2. Plant Material and Growth Conditions

Six barley (*Hordeum vulgare* L.) genotypes used in this study (Table 1) include germplasm obtained from ICARDA (27 barley entries from the Barley Observation Nursery (selected from 328 entries), specifically 5 entries from the Heat Nursery Q2-4 (selected from 458 entries) and 11 entries from the Special Heat Nursery (selected from 320 entries), evaluated during the cropping cycle (1999–2003) [30]. A few lines are among the best lines selected from a set of Omani Batini barley landrace from 2308 subpopulations (Batini 1-7 and 1-5) evaluated by Jaradat et al., [27,28] for tolerance to different levels of continuous salinity during germination and seedling growth attributes.

**Figure 2:**
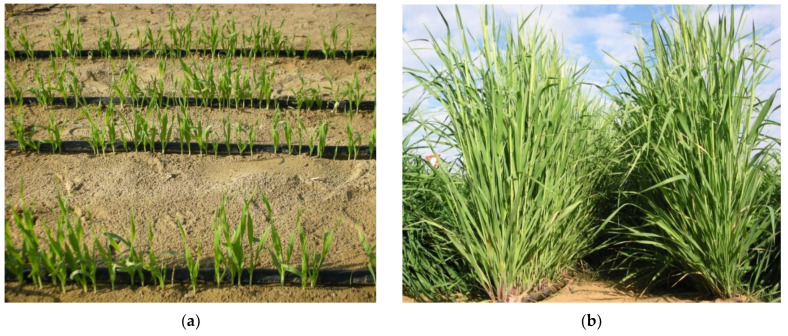

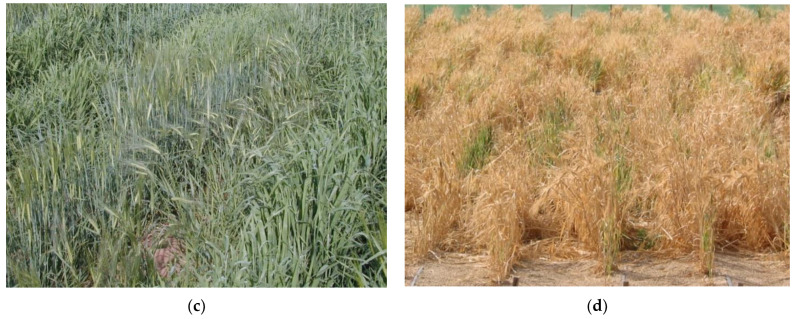
(**a**) Barley field plots for sustainable crop production in sandy marginal hyper-arid desert soils at ICBA, Dubai, UAE. (**b**) Irrigation systems, seedling growth, tillering and spike development. (**c**) Barley crop at grain filling stage. (**d**) Barley crop at maturity stage.

The field plot was prepared by harrowing 1–2 times, followed by planking. Organic fertilizer (N 1.5, K 1.65 and Na 1.22%; pH 7.7, C:N ratio 16.5, organic matter 41% and moisture 1.64%) was applied (30 t ha^−1^) at the surface before soil was incorporated. The seeds (1600 per each genotype) of each individual barley line were sown (2 November 2013) manually in the rows (0.5 m spacing) in the field with a plot size of 2 m × 4 m (plot area of 8 m^2^). The experimental design was a RCBD split plot with three replications. The main-plot factor was the salinity level (0, 7 dS m^−1^, 14 dS m^−1^) and the subplot factor was the genotypes that were randomized within each main-plot. The target salinity was maintained throughout the cropping season and a portable EC meter was used to monitor the salinity twice a week. The crop was irrigated using a drip irrigation system, spreading on the soil surface, having a 4 L hr^−1^ flow rate. A distance of 0.5 m was maintained between rows while the drippers were 0.25 m apart (Figure 2). The irrigation period was variable and depended upon the climatic conditions and crop development stage, ranging from full tillering to dough making. The irrigation program was established so that the plant receives total irrigation (net irrigation + effective rainfall) of around 80% crop evapotranspiration (ETc) plus 20% leaching requirement. During the grain filling period, a net (mesh size of c. 15 × 15 mm^2^) was used to prevent the entry of small birds and to save the grain losses. The impact of saline water treatments (0, 7, 14 dS m^−1^) on growth attributes, stable isotope composition of carbon and nitrogen, leaf ion homeostasis, yield components, harvest index, grain protein contents, and yield stability was evaluated on a selected set of 6 barley genotypes (Batini landraces, varieties, and heat nurseries) (Table 2).

### 2.3. Growth, Agro-Morphological, Leaf Ion Homeostasis and Yield Traits Measurements

From each subplot, the whole plant was harvested from the middle 1 m of two central rows and data were recorded for different agronomical traits (growth, number of tillers, plant biomass), yield traits (number of spikes, number of grains/spike, grain yield, harvest index), and biochemical attributes (Na^+^, Cl^−^, K^+^). The samples were collected to measure fresh biomass (FW) and dry biomass (DW) after the plant samples were dried at 70 °C for 72 h. Briefly, the dried leaves were ground into a fine powder and then ashed for 6 h at 550 °C. After that, 2 N HCl was added to the cooled ash, and the solution was filtered and tested after 15 min. Inductively coupled plasma optical emission spectrometry (Perkin Elmer Optima 4300DV) was used to determine the concentrations of different elements and expressed as mg/100 g dry weight (DW) [31].

### 2.4. Harvest Index (%)

The harvest index was calculated by using the following formula.

Harvest index (%) = Grain yield/dry biomass × 100(1)

### 2.5. Grain Yield

A sample line of 1 m length was harvested, and seeds were removed from the panicle of plants/plot, threshed, weighed (g m^−2^), then converted into t ha^−1^.

### 2.6. Stable Carbon and Nitrogen Isotope Analysis

The leaf samples from each treatment and control were collected, oven dried, and ground into a fine powder. Total N and C contents (% dry matter) were measured by elemental analysis (Flash EA-1112, Swerte, Germany). Dry ground plant material was weighed (1700–2100 µg) using a high precision analytical balance (Metler Toledo GmbH, Greifensee, Switzerland), and filled in tin capsules (5 × 3.5 mm, Elemental Microanalysis Limited, Okehampton, UK). Tin capsules (pressed are in the shape of a microball) were combusted (1600–1800 °C) using an automated elemental analyser coupled to an Isotope Ratio Mass-Spectrometer (Finnegan: Thermo Fisher Scientific, model MAT-253, Swerte, Germany). The Isotopic Ratio Mass Spectrometer has an analytical precision better than 0.3‰ for ^15^N and 0.05‰ for ^13^C.

Carbon and nitrogen isotope compositions were calculated as:

δ (‰) = [(R_sample_/R_standard_) − 1)] × 1000 (2)

where R_sample_ is the ratio of ^13^C/^12^C or ^15^N/^14^N, and R_standard_ were the standards used. Atmospheric N_2_ was the standard for nitrogen while Vienna PeeDee Belemnite (VPDB) was the standard for carbon. The accuracy and reproducibility of the measurements of δ^13^C and δ^15^N were checked with an internal reference material (NBS 18 and IAEA-C6 for C), and (IAEA-310A and IAEA-N1 for N), and acetanilide for C/N% ratios, respectively.

Carbon isotope discrimination is a measure of the carbon isotopic composition in plant material relative to the value of the same ratio in the air on which plants feed:

Δ (‰) = [(δa − δp)/(1 + δp)] × 1000(3)

where Δ represents carbon isotope discrimination, δa and δp refer to δ^13^C of air CO_2_ and plant material, respectively.

Farquhar et al. [32] and Farquhar and Richards [33] indicate that carbon isotope discrimination in leaves of plants can be expressed in relationship to CO_2_ concentrations inside and outside of leaves in its simplest form as:

Δ = *a* + (*b* − *a*) *C*i/*C*a

Δ = 4.4 + (27 − 4.4) *C*i/*C*a (4)

where *a* is discrimination that occurs during the diffusion of CO_2_ through the stomata (4.4‰), *b* is discrimination by RuBisCO (27‰), and *C*i/*C*a is the ratio of the leaf intercellular CO_2_ concentration to that in the atmosphere *C*i/*C*a- ratio of intercellular to atmospheric CO_2_ concentration. Equation (4) establishes a direct and linear relationship between Δ and *C*i/*C*a. Therefore, the measurement of Δ gives an estimation of the rate-weighed value of *C*i/*C*a.

#### Intrinsic Water Use Efficiency (iWUE)

The term “intrinsic water-use efficiency” can be defined as the ratio of the instantaneous rates of CO_2_ and transpiration at the stomata. Intrinsic water use efficiency (iWUE) was calculated according to the following equation:

iWUE = A/g = *C*a [1 − (*Ci/Ca*)] × (0.625) (5)

where A is the rate of CO_2_ and “*g*” is the stomatal conductance. 

Carbon isotope discrimination (Δ^13^C), the ratio of the leaf intercellular CO_2_ concentration to that in the atmosphere (*C*i/*C*a), and intrinsic water use efficiency (iWUE) were determined according to the theory documented by Farquhar et al. [32] and Farquhar and Richards [33]. The close relationship between Δ^13^C and *C*i/*C*a has been explained on the basis that the observed differences reflect the variation of *C*i/*C*a in the carboxylation step of photosynthesis, in response to environmental constraints that affect stomatal regulation. Both *C*i/*C*a and iWUE were derived from δ^13^C basic data using Equations (4) and (5) as reported previously [34,35,36].

### 2.7. Grain Protein Contents Measurements

From each barley genotype, 200 mg FW (three replicates/treatment) were employed for the quantification of grain protein contents using commercial bovine serum albumin (BSA) through Bradford assays [37], as reported previously [38].

### 2.8. Statistical Analysis

Experiment field data were analyzed through SPSS (version 19.00) using a general linear model. The differences between treatment means, genotypes, and their interaction were determined using Tukey’s test (*p* ≤ 0.05). The yield stability of different genotypes at different levels of salinity was computed through static yield stability index (*S*^2^i) and dynamic yield stability index (*W*^2^i) [39,40] as reported previously [41]. 

## 3. Results

### 3.1. Impact of Salinity Treatments and Genotypes on Growth Attributes

The present study assessed whether barley could be extended as a crop to more salt-degraded marginal sandy areas in UAE by irrigating with low quality saline water (E_C_ = 7 and 14 dS m^–1^). Soil biochemical analysis showed that the soil is sandy loam type. The soil samples showed that soil had low organic matter (OM) content (Table 2) and low contents of nitrogen, phosphorus, and potassium. Mean squares for number of tillers, spike numbers, grain yield, and harvest index were significant (Table 3). The results of the present study demonstrate that both water salinity levels and genotypes in each assessment act independently on the above mentioned attributes. The environmental data (temperature, humidity, and evapotranspiration) during the study period 2013–2014 are shown in Figure 1.

### 3.2. Effect of Salt Stress on Morpho-Physiological Characteristics

Salt water significantly affected the plant dry biomass (PDB) due to irrigation water salinity at all levels. Saline water treatments caused a reduction in PDB from 16% to 31% at 7 and 14 dS m^−1^ respectively (Table 3). Barley genotype 113/1B (of Batini) produced the highest plant dry biomass (116.2 t ha^−1^), followed by N1-29 and 59/3A (110.3 and 109.1 t/ha). The lowest PDB was produced by Barjouj (105 t ha^−1^) (Table 4). In addition, the number of tillers m^−2^ significantly reduced following exposure to severe salt stress (572.9) as compared to control (700.5). The percentage reduction in the number of tillers m^−2^ was 18–10% from 14 to 7 dS m^−1^ NaCl stress. Physiological traits, e.g., number of spikes m^−2^, were also decreased at each salinity stress and the highest reduction (20%) was observed at 14 dS m^−1^ NaCl, respectively, compared to the non-saline treatment (Table 3). Barley genotypes 113/1B, 59/3A, and Alanda01 exhibit the highest tillers m^−2^, namely 681.1, 635.1, and 616.4, respectively. However, barley genotype N1-29 exhibits the smallest tillers m^−2^ (606) as compared to other genotypes. There was significant variation in the production of the number of spikes^−2^. The number of spikes m^−2^ was highest in barley variety Alanda01 (620.8), followed by 113/1B (593), while the lowest number of spikes m^−2^ (556.2) was exhibited by Barjouj, respectively (Table 4). Genotype Alanda01 exhibited the highest grain numbers/plant (527.9) followed by 113/1B (508.4) while the lowest grains/plant was produced by Barjouj (480.5) (Table 4). A similar pattern of variation was obtained for number of grains/spike in the corresponding barley genotypes.

### 3.3. Leaf Mineral Analysis

The concentrations of Na^+^ and Cl^−^ ions were significantly higher in the barley leaves grown under saline water irrigation compared to control (Table 5). However, the use of saline water also significantly increased the K^+^/Na^+^ ratio in the leaf tissues (Table 4). The K^+^ content was higher with saline water, while the rest of the elements did not show any changes. A significant difference was observed regarding Na^+^ and K^+^ concentrations among the barley genotypes (Table 6). Genotypes Barjouj and N1-29 showed the highest grain yield among the salinity treatments as compared to control and at the same time also accumulated higher K^+^ levels. It was also noticed that these genotypes have a substantial amount of Na^+^ in the leaf tissue that might counterbalance the toxicity effect through the accumulation of K^+^ ions. Potassium concentrations varied widely, 2.6-fold, ranging from 599.4 to 639.2 mg/100 g DW. Sodium concentration also varied from 435.9 to 924.3 mg/100 g DW. Genotypes significantly differed for all traits, including Cl^−^ ions concentration that was significantly higher in Barjouj while the lowest was observed in N1-10 (Table 6). Overall, “N1-10” was the genotype with the highest K^+^/Na^+^ ratio, followed by N1-29, while Barjouj and Alanda01 exhibit the smallest K^+^/Na^+^ ratio among all the barley genotypes.

### 3.4. Effect of Salt Stress on Carbon (C%) and Nitrogen (N%) and C:N Ratios

The level of carbon was reduced at all salinity levels other than control (Table 5). In contrast to C contents, the nitrogen level was elevated at all salty water concentrations. Genotype 113/1B and Barjouj exhibited the highest N% and it was significantly higher than all other genotypes (Table 6). The C% was higher in three barley genotypes, 113/1B, N1-10, and Barjouj, respectively. There was not much difference in the leaf C% among the rest of the barley genotypes (59/3A, N1-29, Alanda01) that exhibit around 27.9%. The C:N value was lowest in 113/1B and Barjouj genotypes while a higher C:N ratio was obtained in 59/3A, N1-10, and N1-29 (Table 6).

### 3.5. Effect of Irrigation Water Salinity, and Genotype on Grain Yield, Stable Isotope Composition of Carbon and Nitrogen

The water salinity generally decreased grain yield among all the genotypes (Table 4). The ANOVA conducted for the carbon isotope data indicated that the Δ values differed among varieties (*p* ≤ 0.05). Most of the varieties provided higher dry matter, and grain yield showed, in most cases, higher Δ values. There was a significant reduction in grain yield that decreased from 62.6% and 48.9% following 20 and 10 dS m^−1^ salt water irrigation, respectively, compared to the control (Table 7). In this context, harvest index (HI) values were reduced following increasing salinity level. HI (%) was decreased by 14% and 9.86% at 20 and 10 dS m^−1^ salinity, respectively, as compared to control (Table 7). Genotypes Barjouj and Alanda01 exhibit higher grain yield (3.96 and 3.87 t ha^−1^), respectively, followed by N1-10 (2.88 t ha^−1^), than all other genotypes. The lowest yield was produced by 59/3A (2.31 t ha^−1^), which was 42% less than the salt tolerant genotype Alanda01 (Table 8). Genotypes Barjouj and Alanda01 exhibit higher HI (36.6%, 36.2%), followed by N1-10 (26.8%), while the lowest HI was observed in 59/3A (20.8%) (Table 8).

The δ^13^C was less negative (−25.5‰) and (−24.7‰) after treatment with saline water (14 and 7 dS m^−1^) as compared to control (−28.88‰), respectively. Genotypes 113/1B and Barjouj demonstrate the highest negative δ^13^C values (−26.49‰, −27.10‰), followed by 59/3A (−25.63‰) Alanda01 (−26.45‰), while the smallest values were obtained in N1-29 (−21.63‰) under salt stress condition. N1-29 showed the lowest negative value of δ^13^C (−21.63‰). The carbon isotope discrimination (Δ^13^C) values were higher in 113/1B and Barjouj (19.6‰ and 19.0‰), while the lowest Δ^13^C values were observed in N1-29 (13.9‰), respectively. A significant difference (5.7‰) (*p* > 0.05) was observed after salinity treatment in carbon isotope discrimination (Δ^13^C), that was in the range of 13.9–19.6‰. Genotypic differences for δ^15^N traits were also examined for salinity treatment, proving higher in treated plants (4.5‰ and 4.8‰) than control treatments (3.3‰). There was not much difference in the barley genotypes for nitrogen isotope composition, which was in the range of 4.4–4.6‰ in most of the genotypes, while Alanda01 exhibit low δ^15^N (3.13‰) values as compared to other genotypes. The leaf N concentration has significant G × T interaction and the δ^15^N of tolerant genotypes was reduced to a greater extent than sensitive ones at all salinity stress, thus causing a significant G × T interaction (Table 8).

The ratio of intercellular to ambient CO_2_ concentration (*C*i/*C*a) was significantly less (0.56 and 0.60) after treatment with 7 and 14 dS m^−1^ as compared to control (0.0.59), indicating the closing of stomata and inhibition of CO_2_ (Table 7 and Table 8). The maximum value of *C*i/*C*a was observed in genotype Barjouj (0.67), followed by Alanda01 (0.64), 113/1B (0.64), and 59/3 A (0.61), respectively (Table 8). The intrinsic water use efficiency (iWUE) values significantly increase following salinity treatment. A continuous increase in the values of iWUE was observed with increasing level of salinity. Our results revealed that iWUE was increased to 58.45%, and 37.85% at 14 and 7 dS m^−1^ NaCl treatments, respectively, as compared to non-saline condition (Table 7). The maximum values of iWUE were observed in genotype N1-29 (144.5) followed by N1-10 (131.8). The minimum iWUE value was documented in Barjouj (81.4) (Table 8).

### 3.6. Impact of Water Salinity on Protein Content in Barley Genotypes

**Figure 3:**
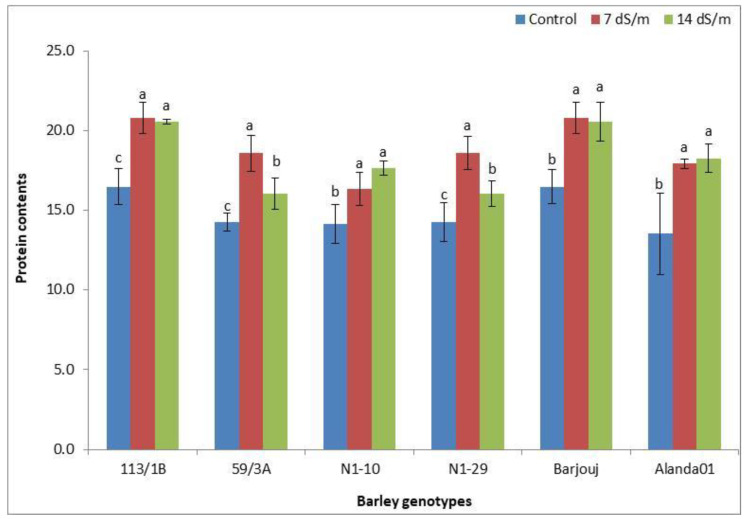
Changes in grain protein contents (mg g^−1^) in 6 barley genotypes following exposure to three different salinity levels (0, 7, 14 dS m^−1^). Each bar represents the mean (±S.E.) of three replicates. Bars with different lower case letters indicate significant difference with respect to control at *p* ≤ 0.05 according to Tukey’s HSD test.

There was a significant impact of saline water stress on the protein contents of barley grains. As compared to control, protein contents in barley grains were enhanced (17.5 and 17.8 mg/g DW) following exposure to both medium and higher salinity. Barley genotypes varied greatly for grain protein contents (Figure 3). GPC was highest in the genotypes 113/1B and Barjouj, ranging from 16.5 to 20.8 mg/g DW. In this regard, the highest GPC was observed in these two genotypes at higher salt stress (14 dS m^−1^). The lowest GPC was observed in genotype Alanda01 (13.5) (Figure 3) in control treatment. GPC ranged from 14.3 to 16.1 mg/g DW, 14.1 to 17.7 mg/g DW, and 14.3 to 18.6 mg/g DW, respectively, in barley genotypes 59/3A, N1-10, and N1-29.

### 3.7. Grain Yield Stability Evaluation

Barley genotypes, varied greatly for mean grain yield across the treatments (mi) (Table 9). The barley genotypes exhibited very different scores for both static environmental variance (*S*^2^i) and dynamic Wricke’s ecovalence (*W^2^i*). The static environment variance for grain yield among the six barley genotypes ranged from 0.122 to 1.031 while Wricke’s ecovalence varied from 0.101 to 1.077. In these stability analyses, the lowest values demonstrate the stability in yield over saline environments. The variety ‘Barjouj’ was static stable and high yielding, ranking first for *S*^2^i grain yield index across all saline environments, and it was followed by Alandra01. The genotype ‘Alandra01’ showed stable mean yield (*W*^2^i) and ranked first among all the genotypes across all environments. Moreover, variety ‘Alandra01’ was static stable (*S*^2^i) and high yielding, ranking second for *W^2^i* grain yield index (Table 9).

### 3.8. Grain Yield Stability Evaluation

The barley varieties, nurseries, and landraces showed higher mean grain yield across the treatments (mi) (Table 9). The barley genotypes exhibited very different scores for both static environmental variance (*S*^2^) and dynamic Wricke’s ecovalence (*W*^2^). The static environment variance for grain yield among the six barley genotypes ranged from 0.122 to 1.031 while Wricke’s ecovalence varied from 0.101 to 1.077. In these stability analyses, the lowest values demonstrate the stability in yield over saline environments. The variety ‘Barjouj’ was static stable and high yielding, ranking 1st for *S*^2^i grain yield index across all saline environments, and it was followed by Alandra01. The genotype ‘Alandra01′ showed stable mean yield (*W*^2^i) and ranked first among all the genotypes across all environments. Moreover, variety ‘Alandra01′ was static stable (*S*^2^i) and high yielding, ranking second for *W^2^i* grain yield index (Table 9).

## 4. Discussion

In hyper arid, salt-degraded, and marginal environments, there are several production constraints that significantly disturb growth, productivity, and crop yield stability. Under the prevailing conditions of the UAE, there is a severe lack of freshwater resources and most of it is only available for domestic purposes and other high value issues. In this situation, the management of available natural water resources (i.e., underground low-quality saline water) and nutrient poor sandy soils, and their conversion to a sustainable production system for food and feed is a most appropriate approach to the rehabilitation of these degraded lands. Soil biochemical analysis indicates that the soil is sandy with almost no organic matter content (Table 3).

Irrigation with saline water decreased the plant dry biomass at all salinity levels, ranging from 16–31%. Meanwhile, genotype 113/1B exhibited the maximum dry biomass (116.2 t/ha) and Barjouj produced the lowest PDB (105 t ha^−1^) (Table 4). In this context, the number of tillers m^−2^ decreased following exposure to higher salt stress and the reduction was 10–18% at 7–14 dS m^−1^ NaCl stress. According to the reports of Arif et al. [42], sodium stress is a serious global concern for sustainable agriculture that disrupts morphological, cellular, and physiological traits, affecting plant growth and development at all stages of development. Physiological traits, e.g., number of spikes m^−2^, were also decreased at each salinity stress and the highest reduction (20%) was observed at 14 dS m^−1^ NaCl, respectively, compared to the non-saline treatment (Table 3). Barley genotypes 113/1B, 59/3A, and Alanda01 exhibit the highest tillers m^−2^ while N1-29 exhibits the smallest tillers m^−2^. There was significant variation in the production of the number of spikes^−2^. The highest number of spikes m^−2^ was obtained in barley variety Alanda01 followed by 113/1B while the lowest number of spikes m^−2^ was exhibited by Barjouj, respectively (Table 4). Genotype Alanda01 exhibited the highest grain numbers plant^−1^ followed by 113/1B while the lowest grains/plant was produced by Barjouj. A similar pattern of variation was obtained for number of grains spike^−1^ in the corresponding barley genotypes.

Understanding the biochemical, morphological, and physiological response mechanisms that play a role in improving adaptation to saline water environments is limited and the development of even more salt tolerant barley cultivars is of vital importance [41,42,43,44,45]. This study investigated the salinity tolerance of genetically diverse barley genotypes and landraces based on agro-morphological, biochemical, physiological, and photosynthetic carbon isotope discrimination attributes in order to identify promising genotypes for salt tolerance screening. The current study showed that salt stress reduced PDB from 16% to 31% in field plots that received highly saline water (14 dS m^−1^)(Table 3). Barley genotype 113/1B showed higher dry biomass while Barjouj exhibited the lowest PDB (Table 4). Morpho-physiological traits varied among barley genotypes due to genotypic differences, differences in saline environment, and also genotype by environment interactions. It is critical to understand the scope of such variations in order to develop breeding strategies and improve selection methods.

Salinity stress can cause inhibition of the photosynthetic process and hence agricultural productivity, yield stability, and environmental sustainability. Plants’ ability to become photosynthetically active in adverse saline conditions, on the other hand, is largely untapped. Salt stress has been shown to reduce barley yield by interfering with reproductive development and grain filling [46,47]. In barley, both successful seed setting and grain filling processes are critical for determining final grain yield. During the growth, reproductive, and grain filling periods, barley genotypes were exposed to salt stress (14 dS m^−1^), with an average of number of spikes m^−2^. However, 113/1B, 59/3A, and Alanda01 showed a greater number of tillers m^−2^ as compared to other genotypes, while genotype N1-29 displayed the lowest tillers m^−2^. We observed a significant reduction in grains per spike and grain weight across genotypes grown under saline conditions, resulting in a reduction in grain yield of 23% on average when compared to non-saline conditions (Table 4). Genotype Alanda01 revealed highest grain numbers plant^−1^ (527.9) followed by 113/1B (508.4) while Barjouj (480.5) produced the lowest grains plant^−1^. Meanwhile, severe salinity stress during the grain filling stage may have an impact on other yield components, such as grain filling duration and grain filling process, and hence can cause significant effects in lowering grain weight and yield in barley [46,47,48].

In response to salt stress, Na^+^ and Cl^−^ levels were significantly higher in the barley leaves while the K^+^/Na^+^ ratio in the leaf tissues increased consistently. The K^+^ content was higher with saline water, while the rest of the elements did not show any changes. The K^+^ levels were consistent with K^+^ availability, even under saline environment, and they could also be linked to the physiological changes seen in barley. Plant exposure to a saline environment can cause higher Na^+^ absorption via roots, which leads to the development of osmotic and water stress [48,49,50]. In comparison to the control, increased salinity levels resulted in an increase in tissue sodium and chloride content. Under severe salt stress, the increase in tissue sodium affects cell wall integrity and cell expansion, in addition to oxidative damage [51]. In this context, Na^+^ stress confines the absorption of other essential nutrient elements (K^+^, Ca^2+^, P, N) [48,52] that trigger the disturbance in the ion homeostasis, physiological, and biochemical cell activities [53].

Genotypes Brjouj and N1-29 showed the highest grain yield among the salinity treatments as compared to control and at the same time also accumulated higher K^+^ levels. It was also noticed that these genotypes have a substantial amount of Na^+^ in the leaf tissue that might counterbalance the toxicity effect through the accumulation of K^+^ ions. Potassium concentrations varied widely, 2.6-fold, ranging from 599.4 to 639.2 mg/100 g DW. Similar genotypic variation for salinity stress tolerance was demonstrated in barley [54]. Such genotypic variation for salt tolerance might be due to the presence of a discrepancy among physiological traits, such as photosynthetic capacity, ion uptake, and maintenance of plant water status or antioxidant potential [54]. Other researchers also demonstrated that barley exhibits tolerance to medium salinity [55,56]. Our results showed that N concentration increased after salinity treatments. Barley cultivars 113/1B and Barjouj showed highest N% and it was significantly higher than all other genotypes. The C% was higher in three barley genotype, 113/1B, N1-10, and Barjouj, respectively. Several researchers demonstrated that salt stress impedes the plant growth, physiological attributes, and yield contributing factors, such as the number of fertile tillers, grain weight, yield per square meter, and finally grain yield. The carbon metabolism, plant growth, and nutritional deficiency due to excess sodium accumulation in soil and plant tissues will lead to oxidative disorders and lower crop yield [6,7,10,57,58,59].

### Effect of Irrigation Water Salinity, and Genotype on Grain Yield, Stable Isotope Composition of Carbon and Nitrogen

The assessment of stable isotopes of carbon and nitrogen (δ^13^C and δ^15^N) provides a very useful parameter that can help to analyze the impact of the surrounding environment in which the plants are growing. Meanwhile, carbon isotope discrimination can provide an integrated assessment of the stomatal regulation of internal CO_2_ content as well as elaborate C_3_ plant species’ long-term photosynthetic carbon [32,33]. Leaf growth and area development, photosynthesis, and nitrogen use are all closely related to crop yield. Salinity inhibits leaf growth, limiting grain yield and yield characteristics [60]. The current findings show that when salinity increased from 7 to 14 dS m^−1^, grain yield fell, ranging from 24% to 42.10%. Meanwhile, *C*i/*C*a was much lower, indicating that the stomata had closed (Table 8). Stomatal closure can reduce CO_2_ supply to carboxylation sites, lowering the activity of Ribulose-1,5-bisphosphate carboxylase oxygenase (RuBisCO), carbon synthesis, and translocation [32,33]. Higher Δ^13^C is caused by a higher *C*i/*C*a ratio mainly due to a larger stomatal conductance, which can lead to a higher photosynthetic rate and hence a higher yield, i.e., positive relationship between Δ^13^C and yield. Genotypes Barjouj and Alanda01 exhibit higher HI (36.6%, 36.2%), followed by N1-10 (26.8%), while the lowest HI was observed in 59/3A (20.8%) (Table 8). When different barley genotypes were tested for salinity tolerance, they demonstrated better *C*i/*C*a and yield potential, indicating their adaption to the Dubai climate. There was a substantial difference in seed yield and harvest index between different genotypes which could be attributable to genetic differences. Such variances are much more pronounced in genotypes Barjouj and Alanda01, and 59/3A which had grain yield variation of 1.6%. HI (%) was decreased by 14% and 9.86% at 20 and 10 dS m^−1^ salinity, respectively, as compared to control (Table 7). This is due to some genotypes’ superior tolerance to the UAE’s agro-climatic conditions. Genotypes Alanda01 and Barjouj had the maximum photosynthetic CO_2_ rate (*C*i/*C*a), yield, and productivity and were the most suited and well-adapted genotypes for the Dubai marginal soil environment. N1-10 and N-29 had the lowest rates (37% and 30% lower *C*i/*C*a than Barjouj), indicating that they were the least adapted. The *C*i/*C*a ratio of intercellular to ambient CO_2_ concentrations did not differ significantly between the remaining genotypes (113/1B, 59/3A, Barjouj, Alanda01).

Although variation in plant N isotopic composition (^15^N) does not offer a measure of NUE, it can be used to follow N mobility and infer N sources and/or N cycle dynamics in vegetation at the local, community, and landscape scales. The diffusion gradient for CO_2_ into the leaf through the stomata is linked to both the efficiency of water usage (carbon (C) fixed per unit water transpired) and the efficiency of N use (C fixed per unit N absorbed). Plants need the majority of their water to support photosynthesis through transpiration. Photosynthesis accounts for more than half of total leaf N [61], and total leaf N content and photosynthetic capability are frequently associated [62]. If the CO_2_ diffusion gradient steepens, reductions in stomatal conductance (gs) or higher investments in foliar N can result in higher water-use efficiency (WUE), while lower intercellular CO_2_ concentrations can diminish N-use efficiency (NUE) by reducing rates of C fixation per cell. For salinity treatment, phenotypic differences for ^15^N characteristics were also investigated, and they were found to be larger in treated plants (4.5 and 4.8) than in control treatments (3.3). In terms of nitrogen isotope composition, most genotypes were in the range of 4.4–4.6, while Alanda01 had low ^15^N (3.13) values when compared to other genotypes. The leaf N concentration has a substantial G x T interaction, and tolerant genotypes’ ^15^N was lowered to a greater extent than sensitive genotypes under all salinity stress conditions, resulting in a significant GxT interaction (Table 8). Carbon isotope discrimination (Δ^13^C), the difference in ^13^C/^12^C composition between plant C and environmental CO_2_, has frequently been used to estimate WUE. Previous studies have demonstrated negative correlations between Δ^13^C and WUE under a CO_2_ in various species, such as barley, cowpea, and wheat [63,64,65,66]. Following salinity treatment, the intrinsic water use efficiency (iWUE) values dramatically rise. The genotype N1-29 exhibited highest iWUE values, followed by N1-10, while Barjouj demonstrated the lowest iWUE values (Table 8).

## 5. Conclusions

In conclusion, we found that barley genotypes exhibited wide genetic variability at various salinity levels tested under UAE desert conditions. We did not find this surprising as the genetic diversity of barley might occur because of large variation among climate and seasonal characteristics, cultivation history, and intensity of selection pressure. These genotypes can be profitable in marginal areas using low quality saline ground water and, through genotypic/phenotypic trials, can be utilized for the growth and production of barley and for the rehabilitation of UAE marginal lands. Most of the barley genotypes that exhibited higher grain yield showed high Δ^13^C values. Furthermore, stress tolerance indices, static yield stability index, dynamic yield stability index, and physiological characteristics (selective uptake and transport of Na^+^ and K^+^ and plant vigour) helped us in the assessment of salinity tolerance and comparison of yield from different barley genotypes that will further elucidate adaptation strategies for salt-degraded and marginal lands. Furthermore, the dynamics of this study demonstrated no risk of salt accumulation in these sandy soils of Dubai, UAE, suggesting the sustainability of barley production when irrigated with saline water. Therefore, further investigation is required to certify the genetic variability and adaptive mechanisms of barley for enhancing salt tolerance and crop productivity. 

## Figures and Tables

**Table 1:** The Barley GeneBank accession names and entry number in this study.

S.No.	Accessions Name	Collection Type	Entry Code/Pedigree
1	113/1B	Batini	113/1B
2	59/3A	Batini	59/3A
7	N1-10	nurseries	Manitou//Alanda/Zafraa
8	N1-29	nurseries	Rhn-03//L.527/NK1272
17	Barjouj	varieties	Barjouj
18	Alanda01	varieties	Alanda01

**Table 2:** Physical and chemical characteristics of experimental soil.

				Soil Characteristics							
	Sample Location	pHs	E_Ce_ (dS m^−1^)	Total N mg kg^−1^	P mg kg^−1^	K mg kg^−1^	% Organic Matter	Sand (%)	Silt (%)	Clay (%)	Textural Class
Pre-sowing 2013	Control	6.55	1.538	52.62	5.46	79.2	0.83	97.53	2.26	0.2	Sand
Post-harvest (2014)	7 dS/m	7.35	2.04	52	41.51	45.95	1.46	97.6	2.2	0.2	Sand
	14 dS/m	7.89	4.1	51.59	46.74	41.61	1.32	97.6	2.2	0.2	Sand

**Table 3:** Effect of salt stress on biomass and agro-physiological traits, and yield components across 6 barley genotypes.

Salt Stress Level	Plant Dry Biomass (t ha^−1^)	Number of Tillers m^−2^	Number of Spike m^−2^	Number of Grain Spike^−1^	Grain Numbers Per Plant
Control	130.1 a	700.5 a	652.2 a	46.3 a	664.3 a
7 dS m^−1^ Nacl	109.3 b	629.4 b	583.9 b	40 b	482.8 b
14 dS m^−1^ Nacl	89.8 c	572.9 c	519.6 c	34.2 c	357.8 c
Salinity Treatment (T)	**	**	**	**	**
Genotype (G)	**	**	**	**	**
T × G interaction	**	**	**	**	**

Values in a single column sharing the same letter are not significantly different (*p* ≤ 0.05) according to Tukey’s honestly significant difference (HSD) test. (**) are significant at *p* ≤ 0.05 or 0.001, respectively.

**Table 4:** Barley genotype difference in biomass and agro-physiological traits across all salinity treatments.

Genotypes	Plant Dry Biomass (t ha^−1^)	Number of Tillers m^−2^	Number of Spike m^−2^	Number of Grain Spike^−1^	Grain Number per Plant	Grain Protein Content (mg/g DW)
113/1B	116.2 a	681.1 a	593 b	40.9 b	508.4 b	19.3 a
59/3A	109.1 c	635.1 b	577.5 c	39.5 c	497 c	16.3 b
N1-10	107.6 d	608.6 e	573.1 c	39.1 c	490.3 c	16 b
N1-29	110.3 b	606 f	572 c	39 c	491 c	16.3 b
Barjouj	105 e	610.4 d	556.2 d	37.6 d	480.5 d	19.3 a
Alanda01	107.8 d	616.4 c	620.8 a	43.4 a	527.9 a	16.6 b

Genotype means with different letters within a column for a given trait are significantly different at *p* ≤ 0.05) according to Tukey’s honestly significant difference (HSD) test.

**Table 5:** Effect of salt stress on biomass and agro-physiological traits, and yield components across 6 barley genotypes.

Salt Stress Level	K^+^	Cl^−^	Na^+^	K^+^/Na^+^ Ratio	% N	% C	C:N Ratio	Protein
Control	87.7 c	114.5 c	118 c	0.78 b	2.2 a	29.6 a	14.5 a	13.6 b
7 dS m^−1^ Nacl	772.3 b	984.3 b	994.2 b	0.84 b	2.8 a	27.6 b	10.2 b	17.5 a
14 dS m^−1^ Nacl	1024.2 a	1226.1 a	1157.2 a	1.00 a	2.8 a	27.2 b	9.7 c	17.8 a
Salinity Treatment (T)	**	**	**	ns	ns	**	**	**
Genotype (G)	**	**	**	ns	ns	ns	**	ns
T × G interaction	**	**	**	**	ns	ns	**	ns

Values in a single column sharing the same letter are not significantly different (*p* ≤ 0.05) according to Tukey’s honestly significant difference (HSD) test. ns (non-significant), (**) are significant at *p* < 0.05 or 0.001, respectively.

**Table 6:** Barley genotype difference in biomass and agro-physiological traits across all salinity treatments.

Genotypes	K^+^ (mg 100 g^−1^ DW)	Cl^−^ (mg 100 g^−1^ DW)	Na^+^ (mg 100 g^−1^ DW)	K^+^/Na^+^ Ratio	Leaf N%	Leaf C%	C:N Ratio
113/1B	615.1 c	760.3 c	526.1 d	1.0 a	3.0 a	28.9 a	9.6 c
59/3A	599.4 d	741.8 d	557.1 c	0.9 b	2.6 b	27.9 b	11.5 a
N1-10	575.2 e	717.7 e	435.9 f	1.12 a	2.6 b	28.2 a	11.3 a
N1-29	638.6 a	779.0 b	507.2 e	1.08 a	2.6 b	27.9 b	11.5 a
Barjouj	639.2 a	784.7 a	924.3 a	0.66 c	3.1 a	28.9 a	9.6 c
Alanda01	620.8 b	760.5 c	899.7 b	0.66 c	2.6 b	27.8 b	10.8 b

Genotype means with different letters within a column for a given trait are significantly different at *p* ≤ 0.05) according to Tukey’s honestly significant difference (HSD) test.

**Table 7:** Genotype and treatment effects on seed yield, harvest index, carbon and nitrogen isotope attributes of six barley genotypes grown under different water salinity levels.

Treatments	Grain Yield (t ha^−1^)	Harvest Index (%)	δ ^13^C	Δ^13^C	*C*i/*C*a	iWUE	δ N^15^	Protein
Control	3.8 a	29.4 a	−25.3 a	17.8 a	0.59 a	102.3 a	3.3 c	13.6 b
7 dS m^−1^ Nacl	2.89 b	26.5 b	−24.7 a	17.1 a	0.56 a	109.4 a	4.5 b	17.5 a
14 dS m^−1^ Nacl	2.2 c	25.3 c	−25.5 a	17.9 a	0.60 a	99.9 a	4.8 a	17.8 a
Salinity Treatment (T)	**	**	ns	ns	ns	ns	**	**
Genotype (G)	**	**	ns	ns	ns	ns	**	ns
T × G interaction	**	**	ns	ns	ns	ns	**	ns

SY, Seed yield (t ha^−1^); HI, harvest index (%); *C*i/*C*a, ratio of intercellular to ambient CO_2_ concentration; iWUE, intrinsic water-use efficiency; δ^13^C, stable carbon isotope composition (‰); Δ^13^C carbon isotope discrimination (‰); SY, seed yield (t ha^−1^); δ^15^N, stable nitrogen isotope composition. Values in a single column sharing the same letter are not significantly different (*p* ≤ 0.05) according to Tukey’s honestly significant difference (HSD) test. ns, (**) are non-significant or significant at *p* ≤ 0.05 or 0.001, respectively.

**Table 8:** Genotype and treatment effects on seed yield, harvest index, carbon and nitrogen isotope attributes of six barley genotypes grown under different water salinity levels.

Genotypes	GY (t/ha)	HI	δ^13^C	Δ^13^C	*C*i/*C*a	iWUE_T_	δ^15^N	Protein
113/1B	2.50 c	21.4 c	−26.49 a	19 a	0.64 b	88.4 d	4.6 a	19.3 a
59/3A	2.31 d	20.8 d	−25.63 b	18.1 b	0.61 b	98.4 c	4.4 a	16.3 b
N1-10	2.88 b	26.8 b	−22.73 c	15.1 c	0.47 c	131.8 b	4.4 a	16.1 b
N1-29	2.49 c	21.9 c	−21.63 d	13.9 d	0.42 d	144.5 a	4.4 a	16.3 b
Barjouj	3.87 a	36.2 a	−27.10 a	19.6 a	0.67 a	81.4 e	4.6 a	19.3 a
Alanda01	3.96 a	36.6 a	−26.45 b	18.9 b	0.64 b	88.9 d	3.13 c	16.6 b

SY, Seed yield (t ha^−1^); HI, harvest index (%); *C*i/*C*a, ratio of intercellular to ambient CO_2_ concentration; iWUE, intrinsic water-use efficiency; δ^13^C, stable carbon isotope composition (‰); Δ^13^C carbon isotope discrimination (‰); SY, seed yield (t ha^−1^); δ^15^N, stable nitrogen isotope composition. Values in a single column sharing the same letter are not significantly different (*p* ≤ 0.05) according to Tukey’s honestly significant difference (HSD) test.

**Table 9:** Environmental variance (*S*i^2^) and Wricke’s ecovalence (*W*i^2^) over the saline treatment for the 6 barley genotypes with highest averaged mean yield across treatments (mi).

S.No.	Accessions Name	Collection Type		mi	Si^2^	Wi^2^
1	113/1B	Batini Landraces	LR	2.533	0.912	0.222
2	59/3A	Batini	LR	2.431	0.538	1.077
7	N1-10	nurseries	NS	2.458	0.542	0.154
8	N1-29	nurseries	NS	2.353	1.031	0.717
17	Barjouj	varieties	VT	3.118	0.122	0.111
18	Alanda01	varieties	VT	3.058	0.349	0.101

VT: varieties; NS: Nurseries; LR: Batini landrace.

## Data Availability

All data generated or analyzed during this study are included in this published article.
